# Supplementary material for: Dynamic transcriptomic profiles of zebrafish gills in response to zinc supplementation
Source: BMC Genomics. 2010 Oct 11;11:553. doi: 10.1186/1471-2164-11-553 (PMC3091702; doi:10.1186/1471-2164-11-553)
Supplement: Additional file 2 — Interactive Direct Interaction Network representing the molecular interactions between zinc, copper, iron, calcium and proteins encoded by transcripts changed by zinc supplementation. Mini web-site containing index.html and hyperlinked pages in subdirectory describing a Direct Interaction Network automatically generated based on curated interactions contained within the proprietary PathwayArchitect database. Ovals represent proteins and the circles symbolize metal ions. Objects are coloured by their abundance in zebrafish at the time-point they were significantly different from the control is a scale from -4 fold (dark green) to +4 fold (dark red). Where significant differences were found at more than one time-point, the colour overlay shows expression at the first instance. Dark blue squares denote 'binding', and light blue squares 'expression'; green squares stand for 'regulation', green diamonds for 'metabolism', and green circles for 'promoter binding'. Arrow heads indicate directionality of the interaction where annotated. All nodes and edges can be further interrogated by selecting the relative area of the image. [file 1471-2164-11-553-S2.zip › PathwayArchitect Zn xs DIN/145651.html]

# PROTEIN: MBD1

|  |  |
| --- | --- |
| Name | MBD1 |
| Type | PROTEIN |
| Description | methyl-CpG binding domain protein 1 |
| Note | DNA methylation is the major modification of eukaryotic genomes and plays an essential role in mammalian development. Human proteins MECP2, MBD1, MBD2, MBD3, and MBD4 comprise a family of nuclear proteins related by the presence in each of a methyl-CpG binding domain (MBD). Each of these proteins, with the exception of MBD3, is capable of binding specifically to methylated DNA. MECP2, MBD1 and MBD2 can also repress transcription from methylated gene promoters. Five transcript variants of the MBD1 are generated by alternative splicing resulting in protein isoforms that contain one MBD domain, two to three cysteine-rich (CXXC) domains, and some differences in the COOH terminus. All five transcript variants repress transcription from methylated promoters; in addition, variants with three CXXC domains also repress unmethylated promoter activity. MBD1 and MBD2 map very close to each other on chromosome 18q21. |
| Alias | PCM1 |
|  | the regulator of fibroblast growth factor 2 (FGF-2) transcription |
|  | Mbd1 |
|  | RFT |
|  | Methyl-CpG binding protein MBD1 |
|  | Cxxc3 |
|  | CXXC3 |


---

|  |  |
| --- | --- |
| GO Component | chromatin |
|  | nucleus |
|  | heterochromatin |


---

|  |  |
| --- | --- |
| GO ID | GO:0005634 |
|  | GO:0003677 |
|  | GO:0003714 |
|  | GO:0006355 |
|  | GO:0000785 |
|  | GO:0008327 |
|  | GO:0006306 |
|  | GO:0006366 |
|  | GO:0016481 |
|  | GO:0046872 |
|  | GO:0006350 |
|  | GO:0008270 |
|  | GO:0000792 |
|  | GO:0016564 |
|  | GO:0003700 |


---

|  |  |
| --- | --- |
| MIM | MIM:156535 |


---

|  |  |
| --- | --- |
| Connectivity | 93 |


---

|  |  |
| --- | --- |
| Entrez ID | 4152 |
|  | 17190 |


---

|  |  |
| --- | --- |
| Agilent ID | A\_52\_P158923 |
|  | A\_23\_P208090 |
|  | A\_23\_P164546 |
|  | A\_24\_P185347 |
|  | A\_53\_P159751 |
|  | A\_24\_P376322 |
|  | A\_14\_P139661 |
|  | A\_51\_P143224 |
|  | A\_14\_P124398 |
|  | A\_53\_P157535 |
|  | A\_14\_P113861 |
|  | A\_53\_P130785 |
|  | A\_52\_P131423 |
|  | A\_53\_P154688 |


---

|  |  |
| --- | --- |
| Cellular Localization | Nucleus |
|  | Chromosome |
|  | Organelle |
|  | Cell |


---

|  |  |
| --- | --- |
| Pathway | Zn xs inventory |
|  | Zn xs DIN |


---

|  |  |
| --- | --- |
| GO Process | negative regulation of transcription |
|  | regulation of transcription, DNA-dependent |
|  | DNA methylation |
|  | transcription |
|  | transcription from RNA polymerase II promoter |


---

|  |  |
| --- | --- |
| UniGene | Mm.22522 |
|  | Hs.405610 |


---

|  |  |
| --- | --- |
| Affymetrix Probeset ID | 104340\_at |
|  | 1417968\_a\_at |
|  | 1430837\_a\_at |
|  | 1430838\_x\_at |
|  | 1437061\_at |
|  | 1453678\_at |
|  | 1555611\_s\_at |
|  | 163253\_at |
|  | 203353\_s\_at |
|  | 208595\_s\_at |
|  | 226862\_at |
|  | 241813\_3p\_at |
|  | 241813\_at |
|  | 41828\_at |
|  | 57263\_at |
|  | 59373\_at |
|  | 87170\_at |
|  | 90441\_at |
|  | g7710136\_3p\_a\_at |
|  | g7710138\_3p\_a\_at |
|  | Hs.214140.0.S1\_3p\_at |
|  | Hs.214140.0.S1\_3p\_x\_at |
|  | Hs.6211.4.S1\_3p\_at |
|  | 110484\_at |
|  | RC\_R43885\_at |
|  | TC34184\_at |
|  | TC35520\_at |
|  | Y10746\_at |


---

|  |  |
| --- | --- |
| GO Function | transcriptional repressor activity |
|  | transcription factor activity |
|  | methyl-CpG binding |
|  | transcription corepressor activity |
|  | DNA binding |
|  | zinc ion binding |
|  | metal ion binding |


---

|  |  |
| --- | --- |
| Nucleotide | NM\_002384 |
|  | AF120981 |
|  | AJ564845 |
|  | NM\_013594 |
|  | AK166042 |
|  | AF120982 |
|  | AK156401 |
|  | AF078833 |
|  | AF078832 |
|  | AK178575 |
|  | AF078831 |
|  | BC047353 |
|  | NM\_015844 |
|  | AK204682 |
|  | BC069837 |
|  | BC033242 |
|  | AF078830 |
|  | AF120980 |
|  | NM\_015846 |
|  | NM\_015845 |
|  | AK007371 |
|  | AK148979 |
|  | AK193128 |
|  | AF072241 |
|  | AK004624 |
|  | NM\_015847 |
|  | AK162858 |
|  | AK032392 |
|  | AK032535 |
|  | AF072240 |
|  | AF120978 |
|  | AF120979 |
|  | Y10746 |


---

|  |  |
| --- | --- |
| Protein | NP\_002375 |
|  | AAH33242 |
|  | AAD51444 |
|  | NP\_056669 |
|  | NP\_056723 |
|  | AAD51443 |
|  | CAA71735 |
|  | AAD48908 |
|  | BAC27914 |
|  | AAH69837 |
|  | BAB23419 |
|  | AAD51445 |
|  | AAC68870 |
|  | NP\_056671 |
|  | Q9Z2E2 |
|  | BAE37086 |
|  | CAD92308 |
|  | AAD51442 |
|  | BAE33700 |
|  | AAD50371 |
|  | NP\_038622 |
|  | Q9UIS9 |
|  | AAC68869 |
|  | NP\_056670 |
|  | BAE38538 |


---

|  |  |
| --- | --- |
| Organism | Mammal |


---

|  |  |
| --- | --- |
| Location | chromosome 18, 18q21 (Homo sapiens) |
|  | chromosome 18, 18 45.0 cM, 18 E2 (Mus musculus) |
|  | 18 45.0 cM (Mus musculus) |


---

|  |  |
| --- | --- |
